# Treatment of Harelip and Cleft Palate

**Published:** 1887-05

**Authors:** C. H. Harroun


### Treatment of Harelip and Cleft Palate.—A Case in Practice.

BY C. H. HARROUN.

In presenting this case in practice for your consideration I am simply carrying out the wishes of eminent men in our profession who contributed so much of interest to the meeting of the Ohio State Dental Society in Toledo last October.

The subject of oral deformities is always interesting, and it matters not how it presents itself for our consideration. Correcting irregularities of the teeth, changing the shape of the maxillaries, thereby presenting to the beholder new features, can never lose its interest to us, who labor to bring about these wonderful changes.

This part of the program was made very interesting by Dr. Keely, of Oxford, and Dr. Talbot, of Chicago, Ill. The very simple manner and comparative ease with which these gentlemen moved teeth back into the arch, or changed the shape of one or both maxillaries into a normal condition, only shows what can be accomplished in other directions when we become interested enough in the case to take hold of it with a will.

It was my pleasure during the discussion of oral deformities to present for examination photographs of a case in practice which I had just completed, viz., a case of harelip accompanied with cleft maxillary, hard and soft palate. Drs. Allport, Hunt, Brophy, Harlan, and others joined me in the wish that the case might be put on record in one of our journals.

The contrasts between the photographs, which were of the same person, separated by years of time, and changed by a surgical operation and a little good prosthetic dentistry, created not a little surprise that such and so good results could be accomplished.

I have requested and received from Dr. Samuel S. Thorn, surgeon, a report of the condition of the patient before the operation was performed, also the history of the operation for closing the defective lips. Previous to the operation the patient was presented to me for any advice or suggestion I might make in advance as to how far our art could be depended upon to aid in the full success of the case, so as to modify if necessary the plan of operation, which was fully explained. I advised that the operation proposed by Dr. Thorn be carried out.

Dr. Thorn has given me the following report:

"Harelip Complicated.—Minnie Clark, aged 12 years, American. Parents without disease or deformity. This harelip was attended by fissuration of the hard and soft palate, extending to and involving the uvula, together with deflection of the right central incisor, lateral being absent, with anterior projection and oblique twist of the intermaxillary bone. Communication between the mouth and nasal cavity wide and free, the vomer warped over to the left, nose flattened and broad at the base. In its consideration with an operation for its removal in view, notice of the retraction of the left, and apparent absence of a considerable portion of the right lip, as well as the osseous and dental deformities, was necessary. The left lip, or side, was free of alveolar attachments; the right adherent, but full and soft. Speech much interfered with, both in pronunciation and articulation. Before any operation was attempted I determined, knowing that you had already had much success in adapting obturators in fissures of the hard palate, to see what help, if any, you could give the patient after an operation by me, provided my success should warrant further effort. Your opinion and advice, so comforting and valuable, were of much benefit toward the successful ending of the case."

"Operation.—St. Vincent's Hospital, Toledo:

"The patient, fully anæsthetized, was held in a semi-reclining position, face turned well to one side to promote the escape of blood. By the aid of the deflected incisors the intermaxillary triangular piece was fractured in its line of adhesion to the jaw and palatine plate, then twisted and pressed back to improve its interior line. This done the teeth were extracted. The right lip next relieved of its alveolar attachments, then two semi-circular incisions were made, one on either side, and as near as could be of equal length, having in view the necessity of keeping sufficiently from the edge as to avoid the thin portion, so as to secure deep, thick edges. These vivifying cuts were extended well up into the nostrils, to prevent after puckering. The cheek next liberated from the anterior wall of the upper jaw sufficiently far back and high up as to permit coaptation of the incised edges without any strain of tissue. These and the semi-circular lines of incision, both of the same length, I look upon as essential to success, not alone to aid in maintaining contact, but to avoid the unsightly notch which so often follows these operations. All tissues being free and abundant by reason of this separation from the jaw, the parts were coapted and held in place by means of silver sutures and one harelip pin. To further aid in supporting the parts, and to improve the appearance of the face as a whole, a large harelip pin was thrust through the nose at the alae from right to left, and the parts drawn together and held. This narrowed very much the nose, improved its shape, supported the parts along the line of attachment, and helped to elongate and force down the lip, thereby making an even and full line along the vermillion border. The parts were further supported by plasters, spectacle-shaped, and sufficiently large to extend back to the ear and well over the cheeks. Before attaching the plasters the cheeks were forced well toward the mesial line.

"Very truly,

"SAMUEL S. THORN,

"66 Lagrange Street."

Having completed this most important part of our work, we must of necessity rest from our labors until our patient has fully matured as to stature, and a realization of her condition which will enable us to proceed with the certainty of success, which is now so much desired.

The operation of the surgeon in closing the defective lip is, in a large majority of cases, judging from those presenting themselves for obturators, very poorly performed. This should not be the case. To the contrary, much thought should be given, and, if possible, a good square lip with full muscular action be the result.

Many a failure on our part to enable our patients to articulate well, after they have been supplied with an obdurator, in my opinion, is due to the fact that they have so little use of the upper lip, which has the appearance of being paralyzed, as well as being very much shortened in the center, thus making it impossible to close the lips in articulating.

Five years have passed since the defective lip was closed, and our patient now presents herself for the part of the work which shall enable her to appear in public without hindrance. In examining the oral cavity, we discover the fact that an enemy has been busy, nearly all of her teeth are decayed, many of them very badly, also that the anterior teeth are not in the proper line to give a pleasing appearance when in conversation. These teeth, the left central, lateral, cuspid, first bicuspid, right cuspid, and first bicuspid, we decide to extract, as it will be impossible to construct anything to fill the vacancy occasioned by the loss of the right central, and laterals caused by the cleft, that will not at once be detected by the casual observer. This being accomplished, we allow the mouth to rest and contract as much as possible and I proceed to restore the remaining teeth by filling in most part with gold, using tin in the aproximal cavities, near the cervical border.

Having accomplished all we can in this direction, I at the same time decided of what shape and material the instrument shall be constructed. In order that this shall be as simple as possible, and easily repaired, and at the same time useful for the purpose for which it was constructed, I decided to construct the instrument of hard rubber, with gold clasps or bands around the molars to sustain the obdurator when in place, the anterior teeth attached forming one piece.

The form I used in this case is that recommended by Dr. Suersen, which was first described with cuts in the REGISTER, and is now copied into all works on oral surgery. This form of an obdurator operates well in many cases, and in some very much better than any form that I can conceive, using flexible rubber instead of hard. The whole piece is of hard rubber, very easily repaired, and not likely to get out of order.

We have now finished our labors, with a very gratifying result. The articulation and general appearance of the person are as perfect as we could wish.

